# A Systematic Machine Learning Framework for Evaluating and Ranking Omics Layers in Cancer Drug Response Prediction

**DOI:** 10.3390/cimb48090897

**Published:** 2026-09-02

**Authors:** Sara Amjad, M. M. Sufyan Beg, Mohd. Azhar Aziz

**Affiliations:** 1 Department of Computer Engineering, Aligarh Muslim University, Aligarh 202002, Uttar Pradesh, India; mmsbeg@hotmail.com; 2 Interdisciplinary Nanotechnology Center, Aligarh Muslim University, Aligarh 202002, Uttar Pradesh, India; 3 Cancer Nanomedicine Consortium, Aligarh Muslim University, Aligarh 202002, Uttar Pradesh, India

**Keywords:** drug sensitivity, multi-omics, cancer cell lines, prediction, clustering, accuracy, precision medicine

## Abstract

This study introduces a top-down framework for evaluating the utility of multi-omics features to predict the response of 309 drugs in cancer cell lines. This was done by taking a multi-omics approach where data from proteomic, transcriptomic, genomic, metabolomic, and miRNA were integrated with drug sensitivity (area under the curve, AUC) data. We performed modular dimensionality reduction using t-SNE (t-distributed Stochastic Neighbor Embedding), followed by K-Means clustering to stratify cell lines into data-driven molecular subgroups, and applied a Random Forest model to refine the drug list, selecting only those with a prediction accuracy exceeding 75%. Our findings show that among the evaluated single-omics features, transcriptomics is the most informative; however, multi-omics integration significantly enhances predictive capability compared to single-omics analysis, with a combination of transcriptomic, proteomic, and miRNA data achieving the best predictive performance across both primary and validation datasets. Cluster analysis showed the importance of well-defined clusters, indicating that while silhouette scores were linked to prediction success, biological variability also played a critical role. This study advances personalized oncology treatment strategies and provides a foundation for future studies focused on ranking omics features based on their predictive capabilities, eventually contributing to better therapeutic outcomes. Predictive performance is used here to evaluate omics feature strength, rather than as an objective to optimize predictive models.

## 1. Introduction

By 2045, cancer incidence is projected to increase by 63% and cancer mortality by 73% [1]. Cancer develops through a complex interplay of genetic factors and mutations [2]. However, studies suggest that genetic factors alone are inadequate to explain the initiation and progression of cancer [3]; moreover, multi-omics approaches provide comprehensive insights, uncovering interactions and regulatory mechanisms that single-omics data might miss. Furthermore, combining multiple omics datasets improves disease subtyping, leading to better diagnoses and personalized treatment strategies [4,5,6]. Correlating molecular alterations in tumors with drug response in cancer cell lines reveals that tissue lineage and specific genetic changes play crucial roles in influencing treatment sensitivity [7]. By integrating different omics layers, researchers can capture the complexity of cancer biology and identify biomarkers that predict how patients will respond to a specific drug [8]. For instance, genomics data can reveal mutations driving cancer progression, while transcriptomics can indicate gene expression changes in response to treatment, and proteomics and metabolomics provide an additional layer of information, leading to the identification of previously unexplored therapeutic targets [9].

Machine learning (ML) plays a crucial role in analyzing multi-omics data for drug response prediction (DRP). By applying ML algorithms to large-scale omics datasets, researchers can uncover complex patterns and relationships that would be impossible to discern manually. Integrating multiple data types enhances the predictive power of ML models [10]. Several studies have focused on developing computational and machine learning approaches for drug response prediction by integrating multiple molecular and pharmacological data sources. For example, Huang et al. developed a multiple-layer cell line–drug response network model for predicting breast cancer drug response using molecular and drug-related information [11], while Wang et al. developed DeepDRK, a deep learning framework that integrates multi-omics and drug-related information for drug response prediction and drug repurposing [12]. However, these studies primarily focus on developing or improving predictive models, whereas the present study focuses on identifying the most informative omics layers and combinations of omics features for drug response prediction.

Clustering has already been used in identifying cancer subtypes [13], which can help DRP. Considering the critical role of multi-omics data, clustering, and ML in capturing predictive signals, we propose a novel framework that identifies the most informative feature and feature set and is capable of ranking omics layers based on their predictive strength.

## 2. Materials and Methods

**Figure 1:**
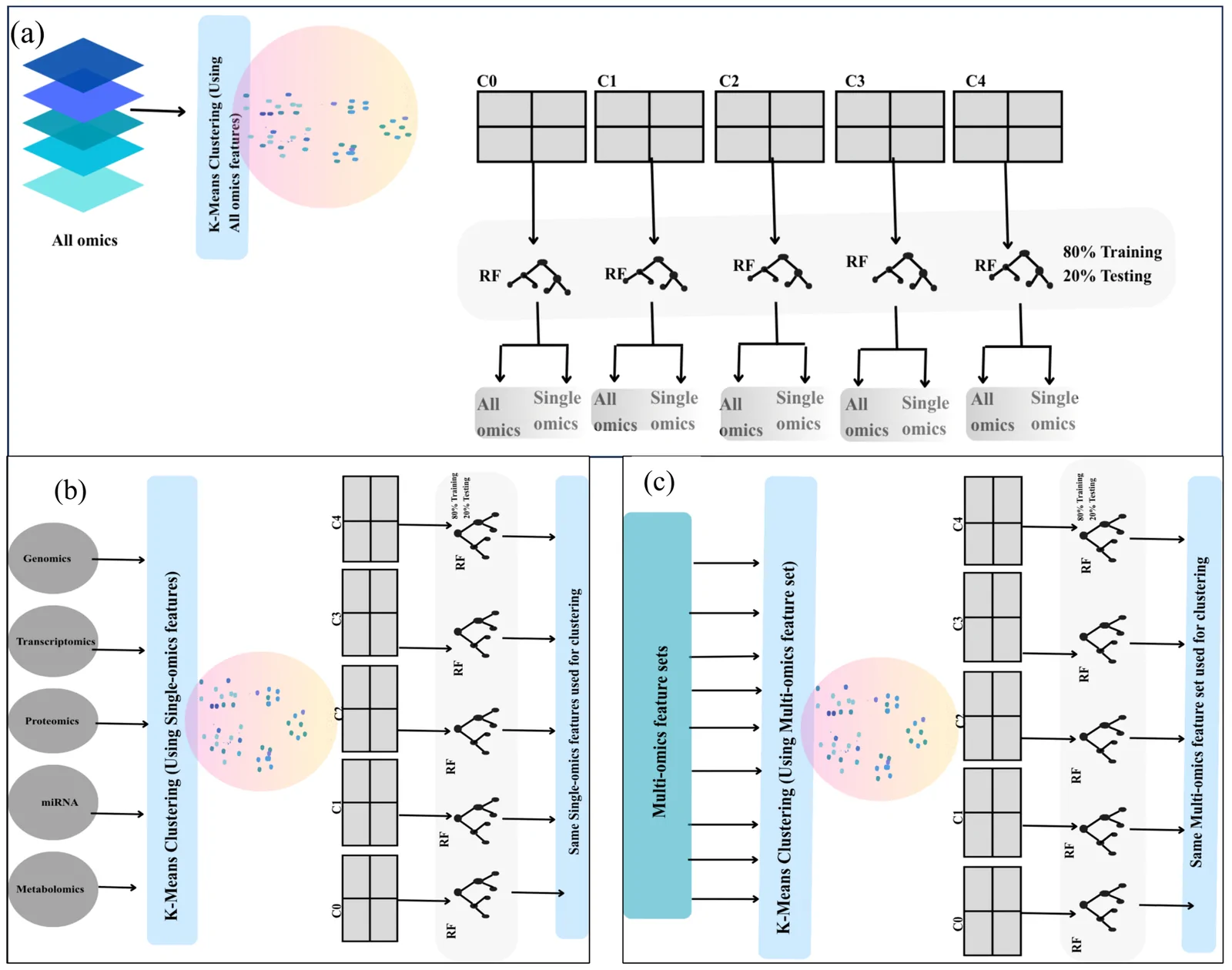
Three-phase framework for multi-omics and single-omics feature strength evaluation. (**a**) In Phase 1, multi-omics data incorporating all omics features are used for clustering. Following this, for each cluster, the all-omics feature set is provided as input to the RFC (Random Forest Classifier) for drug response prediction. Subsequently, individual omics features are also used as input to the RFC for drug response prediction. (**b**) In Phase 2, each single-omics dataset is used both for clustering and drug response prediction. (**c**) In Phase 3, combinations of multi-omics data are used for clustering, followed by drug response prediction using the same combinations of multi-omics.

For better understanding, the entire process is divided into three phases for visual representation. Figure 1 illustrates Phase 1 (Figure 1a), Phase 2 (Figure 1b) and Phase 3 (Figure 1c). Throughout the framework implementation, predictive performance is utilized to evaluate feature strength and is calculated as a trade-off between mean accuracy and the number of drugs with accuracy more than 75%.

### 2.1. Preprocessing

**Figure 2:**
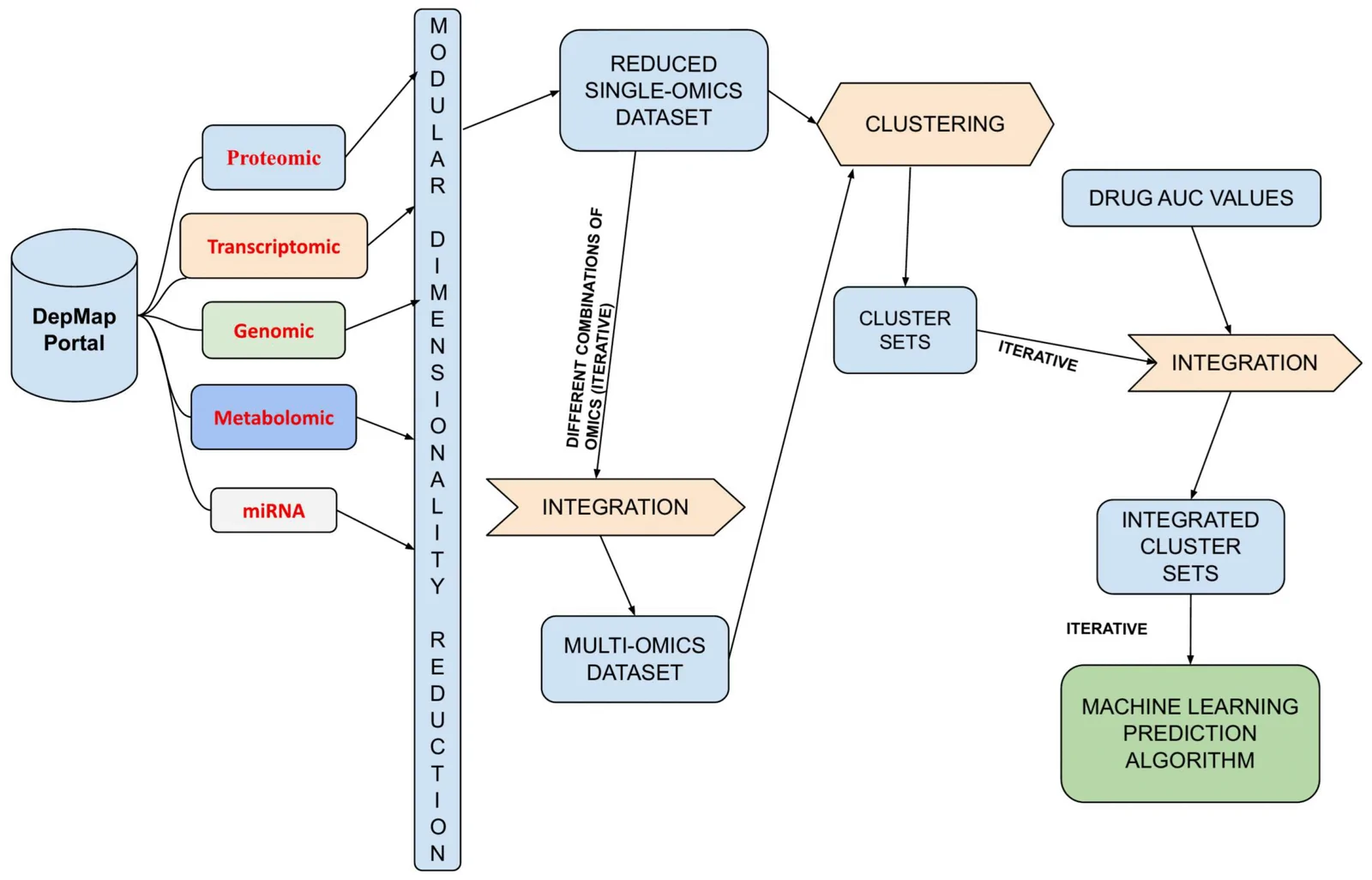
Representation of the preprocessing pipeline for evaluating the strength of multi-omics features in drug response prediction. Illustration of preprocessing steps involved in dimensionality reduction and clustering.

The primary dataset used in this study includes omics data for 585 pan-cancer cell lines and drug sensitivity AUC values for 309 drugs from GDSC1 (Genomics of Drug Sensitivity in Cancer 1) [7], all sourced from the DepMap portal [14]. During initial data quality screening, drug response data were reviewed to ensure adequate availability of AUC values for reliable modeling. The drug set used for analysis was confirmed to have sufficient response coverage across cell lines. Notably, even the drug with the highest level of missingness in the primary dataset had AUC values available for 91 cell lines. In reassessing the preprocessing phase, we also included additional drugs that were missed in our previous work [15]. The omics data were obtained as separate files from the DepMap portal [14], covering various biological dimensions, including proteomic (RPPA-Reverse Phase Protein Array) [16], transcriptomic (gene expression) [16,17], genomic (hotspot mutation and copy number absolute) [16,17], metabolomic [18], and miRNA [16] data. We applied PCA (Principal Component Analysis) for dimensionality reduction in omics features, initially starting with miRNA and metabolomic data using 10 principal components. However, the resulting components failed to retain sufficient local structure (Table S1), leading us to explore alternative approaches. Given that many multi-omics integration methods have not demonstrated significantly better performance than PCA [19] and that PCA-based dimensionality reduction performed poorly on our dataset, we opted for t-SNE (t-distributed Stochastic Neighbor Embedding). This choice was empirically validated through improved clustering performance, particularly for miRNA and metabolomic data (Table S1). The number of t-SNE dimensions was selected empirically based on the resulting clustering performance, with the dimensionality providing better cluster separation retained for subsequent analyses. Transcriptomic, proteomic, copy number aberration, and hotspot mutation datasets were each reduced to three t-SNE features, whereas miRNA and metabolomic datasets were each reduced to two t-SNE features (refer to the Extended Preprocessing section of the Supporting Information for details). The resultant dataset consists of 16 t-SNE-reduced features integrated with AUC values for 309 drugs across 585 cell lines. The preprocessing pipeline has been illustrated in Figure 2.

In addition to the primary dataset, a validation dataset was constructed using the drug sensitivity AUC (PRISM Repurposing Secondary Screen) [20] from the DepMap portal [14]. The final validation dataset consists of 441 pan-cancer cell lines (including 114 unique cell lines that are not part of the primary dataset) and 348 drugs. Notably, the sparsest drug retained AUC values for 177 cell lines. The majority of drugs in the validation dataset are distinct from those in the primary dataset, with only 18 drugs (~5–6%) shared between the two.

### 2.2. Clustering

To identify potential patterns within the integrated multi-omics dataset, we employed the K-Means clustering algorithm. The input to the clustering algorithm is the set of all omics features in the primary dataset. We experimented with several clustering algorithms with different default distance or similarity measures, such as K-Means, which uses Euclidean distance; Spectral Clustering, which uses RBF (Radial Basis Function) kernel affinity; and Affinity Propagation, which uses a similarity matrix based on negative squared Euclidean distance. The best overall results were obtained with K-Means, which achieved an overall silhouette score of 0.170057 and a Davies–Bouldin Index value of 1.854821; Cluster 0 (highest silhouette score cluster for K-Means) achieved a silhouette score of 0.373375 (Table S2). Refer to the ‘Choice of K-Means for Clustering’ section of the Supporting Information for a detailed comparison.

We experimented with different values of k (the number of clusters) while selecting the clustering algorithm and found the best results in terms of silhouette score with k = 5 (silhouette score = 0.373375) in the primary dataset (refer to the Experimenting with k Value section and Table S3 in the Supporting Information for details). For the validation dataset, we employed the same K-Means clustering approach and found the best results with k = 3, where Cluster 0 demonstrated the best separation, with a silhouette score of approximately 0.2. It is important to note that after feature optimization, the silhouette score improved from 0.2 to 0.3 in the validation dataset and from 0.4 to 0.6 in the primary dataset for the best cluster.

### 2.3. Drug Sensitivity Prediction Using Random Forest

We experimented with different ML algorithms, namely Random Forest Classifier (RFC), Support Vector Classifier (SVC), Gradient Boosting Classifier, AdaBoost Classifier, XGBoost Classifier, and Logistic Regression Classifier (LRC), and obtained the best predictive performance with RFC (Table S4). RFC was chosen for DRP based on its robust performance across the various clusters, as illustrated in Table S4 (refer to the Selection of Random Forest section of the Supporting Information for details).

We applied RFC to each cluster individually. The input features for the Random Forest (RF) models comprised the t-SNE-reduced omics data. For drug sensitivity prediction, AUC values were used as the target variables.

We performed (i) handling of missing values, (ii) AUC value mean calculation, and (iii) training (80%) and testing (20%) for each target drug in different iterations, where the number of iterations equals the number of drugs in the dataset (refer to the Implementation of Random Forest Algorithm section in the Supporting Information for details). Cell lines with missing AUC values for a given drug were excluded prior to drug response prediction to avoid biases introduced by response imputation (e.g., mean substitution). While this approach may reduce sample size, it preserves the reliability of training and testing outcomes.

For the validation dataset, RF was selected to maintain uniformity with the primary dataset approach (refer to the Algorithm section in the Supporting Information for detailed steps).

### 2.4. Identification of Best-Performing Single-Omics Feature for DRP

The results from the primary and validation datasets are recorded in Table 1 and Table 2, respectively. We initially used all available omics features as input to ML clustering and prediction algorithms, but later we refined the approach and experimented by changing the inputs for the predictive algorithm from all omics to single-omics. The output for each omics feature is recorded in Table 2 Phase 1. Table 2 Phase 1 comparison with the primary dataset table generated with the same setup is elaborated in the discussion section, as the primary table is part of our previous work [15].

In further refining our approach, we experimented with different single-omics features. We used single-omics features for clustering and applied the same feature for DRP (Table 1 Phase 2 and Table 2 Phase 2). In both the GDSC primary dataset (Table 1 Phase 2) and the PRISM validation dataset (Table 2 Phase 2), transcriptomic data consistently delivers strong performance among the single-omics features, as evidenced by the higher number of drugs with accuracy above 75% and competitive mean accuracies across clusters, as elaborated in the following lines. In the primary dataset, transcriptomics achieves a mean accuracy ranging from 0.80 to 0.86 across clusters, predicting a significant number of drugs with accuracy over 75%, peaking at 42 drugs in Cluster 0, surpassing other omics types. In the validation dataset, while transcriptomics mean accuracy ranges from 0.77 to 0.79, it stands out by achieving the highest number of drugs with accuracy above 75% in Cluster 1 (13 drugs), exceeding most other omics features except miRNA in Cluster 2. Further analysis reveals that miRNA does not perform consistently well in comparison to other single-omics features. For instance, Table 2 Phase 1, which presents results from the validation dataset, shows that miRNA exhibits poor performance, whereas transcriptomics maintains the best performance among single-omics features. Similarly, in Table 1 Phase 2 of the primary dataset, transcriptomics outperforms miRNA.

**Figure 3:**
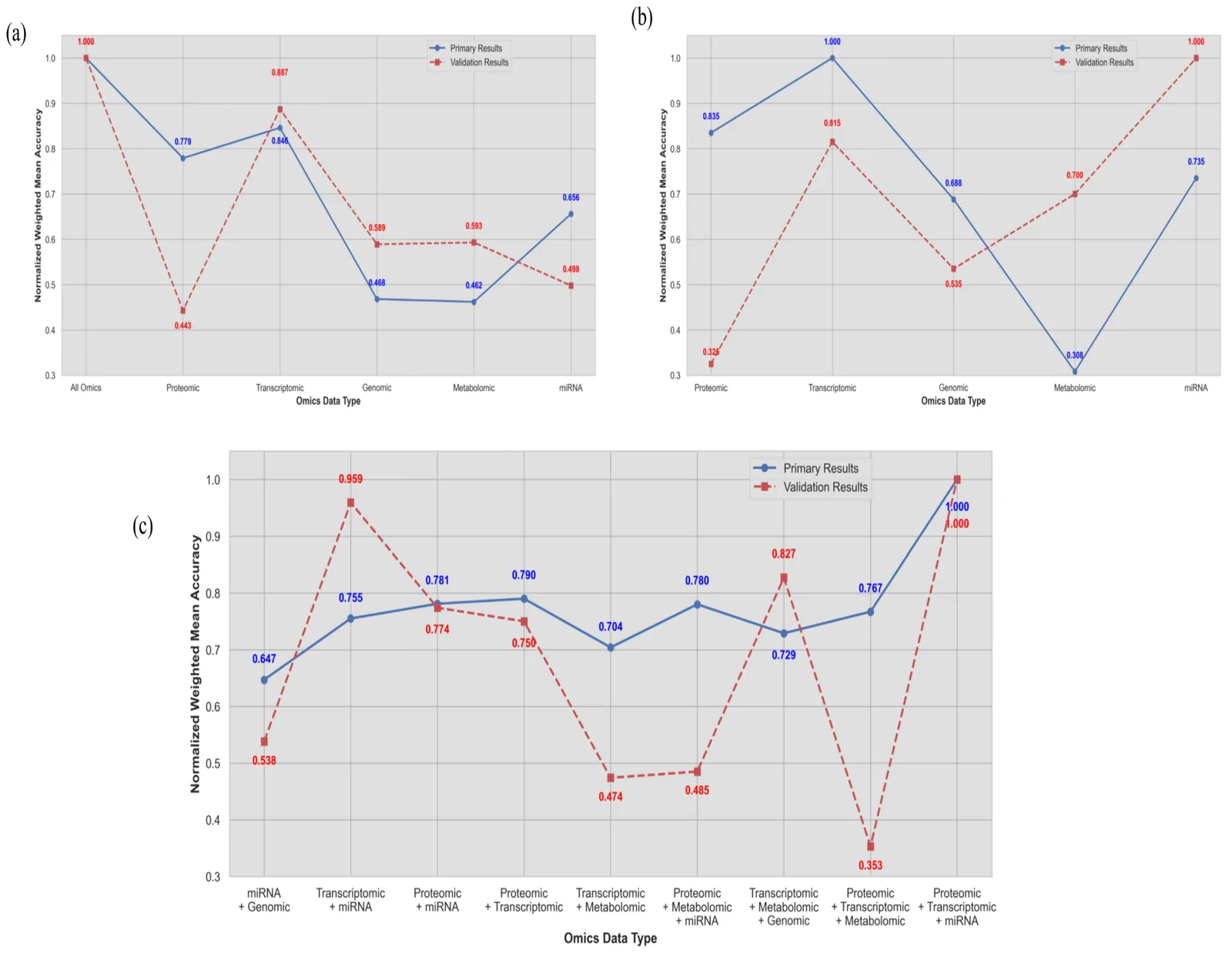
NWMA (Normalized Weighted Mean Accuracy) for different omics features for Phase 1, Phase 2 and Phase 3. Comparison of NWMA across features. (**a**) All omics and single-omics feature of cancer cell lines. The NWMA was calculated using Table 1 [15] and Table 2 Phase 1. (**b**) Single-omics features of cancer cell lines. The NWMA was calculated using Table 1 Phase 2 and Table 2 Phase 2. (**c**) Multi-omics combinations of cancer cell lines. The NWMA was calculated using Table 1 Phase 3 and Table 2 Phase 3.

While transcriptomics does not emerge as the top performer in Table 2 Phase 2 of the validation dataset, it consistently demonstrates strong predictive capability. This trend is more evident in Figure 3a,b, where transcriptomics ranks as the best performer in three out of four cases and the second-best in the remaining one, when compared with other single-omics features. This further corroborates that transcriptomics is the most effective single-omics feature for DRP.

### 2.5. Optimization of Multi-Omics Feature Combinations for DRP

Subsequently, we explored various combinations of multi-omics features by applying the same multi-omics combination for clustering and DRP. The same methodology was applied to both the primary and validation datasets, allowing us to analyze the results and identify common patterns across the datasets. The output results are recorded in Table 1 Phase 3 and Table 2 Phase 3. The combination of transcriptomic + proteomic + miRNA features emerged as the best performer among the 9 multi-omics combinations (Table 1 Phase 3 and Table 2 Phase 3) across both the primary and validation datasets, making it one of the most effective combinations for DRP, as represented in Figure 3c. Refer to the Extended Multi-Omics Selection section of the Supporting Information for tabular analysis. A comparison of the single-omics results (Table 1 Phase 2 and Table 2 Phase 2) with those of the multi-omics results (Table 1 Phase 3 and Table 2 Phase 3), respectively, reveals that optimized multi-omics features yield improved predictive performance.

### 2.6. Handling Clustering and Silhouette Score Analysis

To ensure robust DRP accuracy, we included those omics combination sets in Table 1 Phase 3 and Table 2 Phase 3 where at least one cluster had a silhouette score of ≥0.3 and where the size of all clusters under consideration was above 60. There were a total of 31 combinations of omics features possible; we tested all 31 combinations and found only 5 of them satisfying the criteria in both the primary and validation datasets. Thus, Table 1 Phase 3 and Table 2 Phase 3 contain all five combinations as well as four more that were included intentionally just for analyzing the behavior of clusters with non-qualifying criteria. [Note: When clustering was performed using single-omics for generating Table 1 Phase 2 and Table 2 Phase 2, Genomics did not pass the criteria. Similarly, when clustering was performed using different multi-omics combinations for generating Table 1 Phase 3 and Table 2 Phase 3, the following randomly selected combinations did not qualify but are still included in Table 1 Phase 3 and Table 2 Phase 3 to explore the behavioral dynamics of clusters outside the defined thresholds: miRNA + Genomic, Proteomic + Metabolomic + miRNA, Transcriptomic + Metabolomic + Genomic, and Proteomic + Transcriptomic + Metabolomic].

### 2.7. Mathematical Formulation of Weighted Mean Accuracy (WMA)

To analyze the performance of different omics feature sets, we have derived WMA (Weighted Mean Accuracy). The normalized WMA value validates our visual interpretation. We have represented the normalized WMA values as a line graph in Figure 3. In our study, classification accuracy is used as the standard performance metric for evaluating drug response prediction. The proposed Weighted Mean Accuracy (WMA) is derived from accuracy and is introduced as a complementary aggregation metric, not as a replacement for standard metrics. WMA summarizes mean accuracy across clusters while weighting by the number of high-performing drugs (accuracy > 75%), enabling comparative evaluation of omics feature strength and robustness.

#### 2.7.1. Definition of Weighted Mean Accuracy

Let Mi represent the mean accuracy of cluster i, and let N_i_ denote the number of drugs for which the framework achieves an accuracy greater than 75%. The Weighted Mean Accuracy (WMA) is defined as:

(1) $$WMA=\sum_{i=1}^{k}M_{i}\times N_{i}$$

where

- k is the total number of clusters;
- M_i_ is the mean accuracy of cluster i;
- N_i_ is the number of drugs meeting the performance threshold in cluster i.

Equation (1) ensures that the mean accuracy values are weighted based on the number of drugs contributing to high accuracy, thereby preventing datasets with fewer high-accuracy drugs from disproportionately influencing the final performance score. It also ensures that feature sets predicting a larger number of drugs with high accuracy are appropriately prioritized, preventing overestimation of performance when high accuracy is observed from a limited number of drugs.

#### 2.7.2. Normalization of WMA

WMA values are normalized relative to the highest observed WMA score for each phase in the primary and validation datasets separately, ensuring a range between 0 and 1. The normalized WMA (NWMA) is computed as:

(2) $$NWMA=\frac{{\mathrm{WMA}}_{j}}{{\mathrm{WMA}}_{max}}$$

where:

- WMA_j_ is the computed WMA for omics feature j;
- WMA_max_ is the highest WMA value among all omics datasets in the specific phase.

#### 2.7.3. Rank Analysis

For an omics feature i, let:

- R_primary,i_ be its rank in the primary dataset from Figure 3a,b.
- R_validation,i_ be its rank in the validation dataset from Figure 3a,b.

The Rank Difference Di and Rank Sum Si for each omics feature are calculated as:

(3) $$D_{i}\;=\;\left|R_{\text{primary},i}\;-\;R_{\text{validation},i}\right|$$

(4) $$S_{i}\;=\;\left|R_{\text{primary},i}\;+\;R_{\text{validation},i}\right|$$

where:

- A lower D_i_ value indicates that the feature has a consistent ranking across both datasets.
- A higher D_i_ value suggests a greater discrepancy between primary and validation rankings.
- A lower S_i_ value indicates that the feature is more informative for DRP.
- A higher S_i_ value suggests the feature is less informative for DRP.

Insights from Total Rank Difference and Total Rank Sum

- Most Stable Features: Transcriptomic and Genomic (Total Rank Difference = 1).
- Least Stable Feature: Proteomic (Total Rank Difference = 6).
- Best-Performing Single-Omics Feature: Transcriptomic (Total Rank Sum = 7).
- Worst-Performing Single-Omics Feature: Genomic and Metabolomic (Total Rank Sum = 17).

A detailed comparison of the Rank Difference and Rank Sum values across omics types is shown in Table 3.

Refer to the Weighted Mean Accuracy Section of the Supporting Information for an example.

## 3. Results

Analysis of the outputs generated across different phases in Table 1 and Table 2 shows several consistent trends:

### 3.1. Clustering Improves Performance

**Figure 4:**
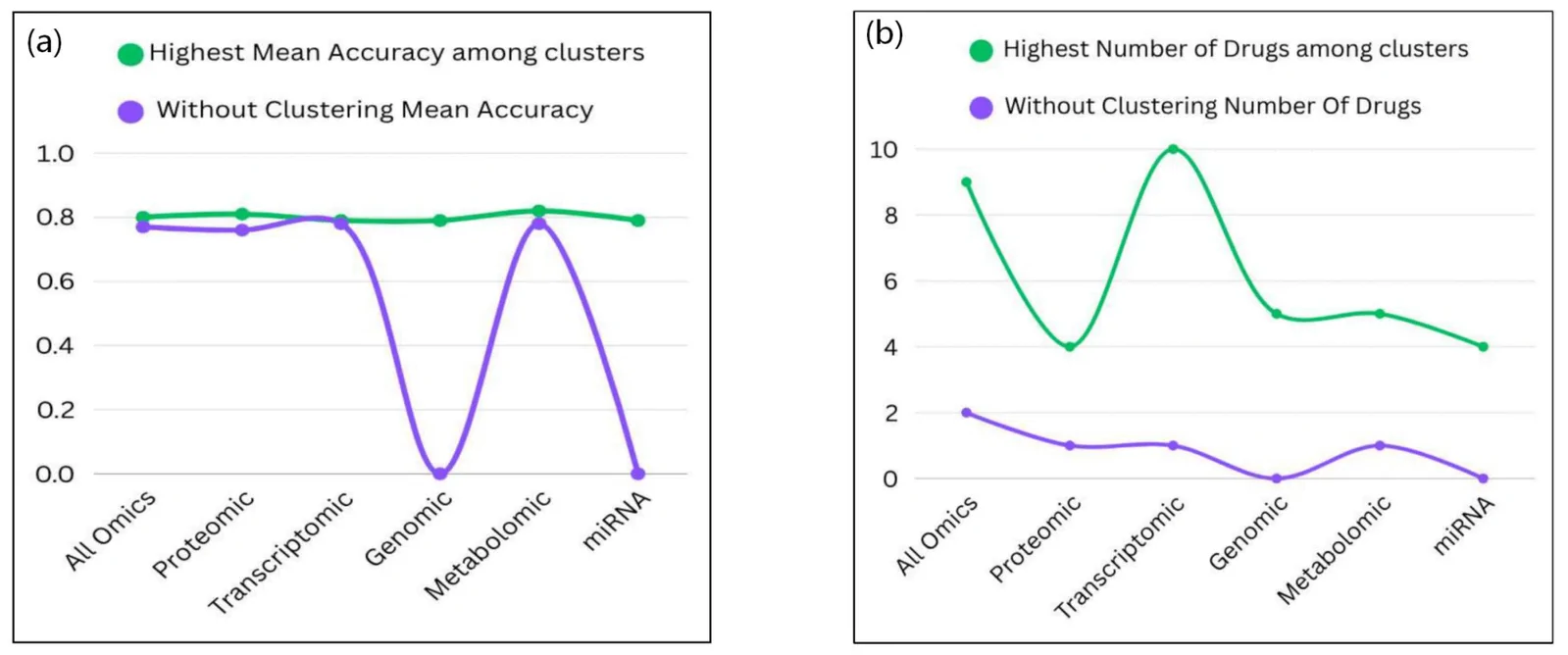
Comparison of clustered and non-clustered predictive performance across different omics features in Table 2 Phase 1. (**a**) Comparison of the highest mean accuracy obtained across clusters for each omics feature with the corresponding mean accuracy obtained without clustering. (**b**) Comparison of the highest number of drugs predicted with accuracy greater than 75% across clusters for each omics feature with the corresponding number obtained without clustering.

This trend is evident in Table 2 Phase 1 and illustrated in Figure 4.

### 3.2. Multi-Omics Show Stronger Predictive Capability than Single-Omics

**Figure 5:**
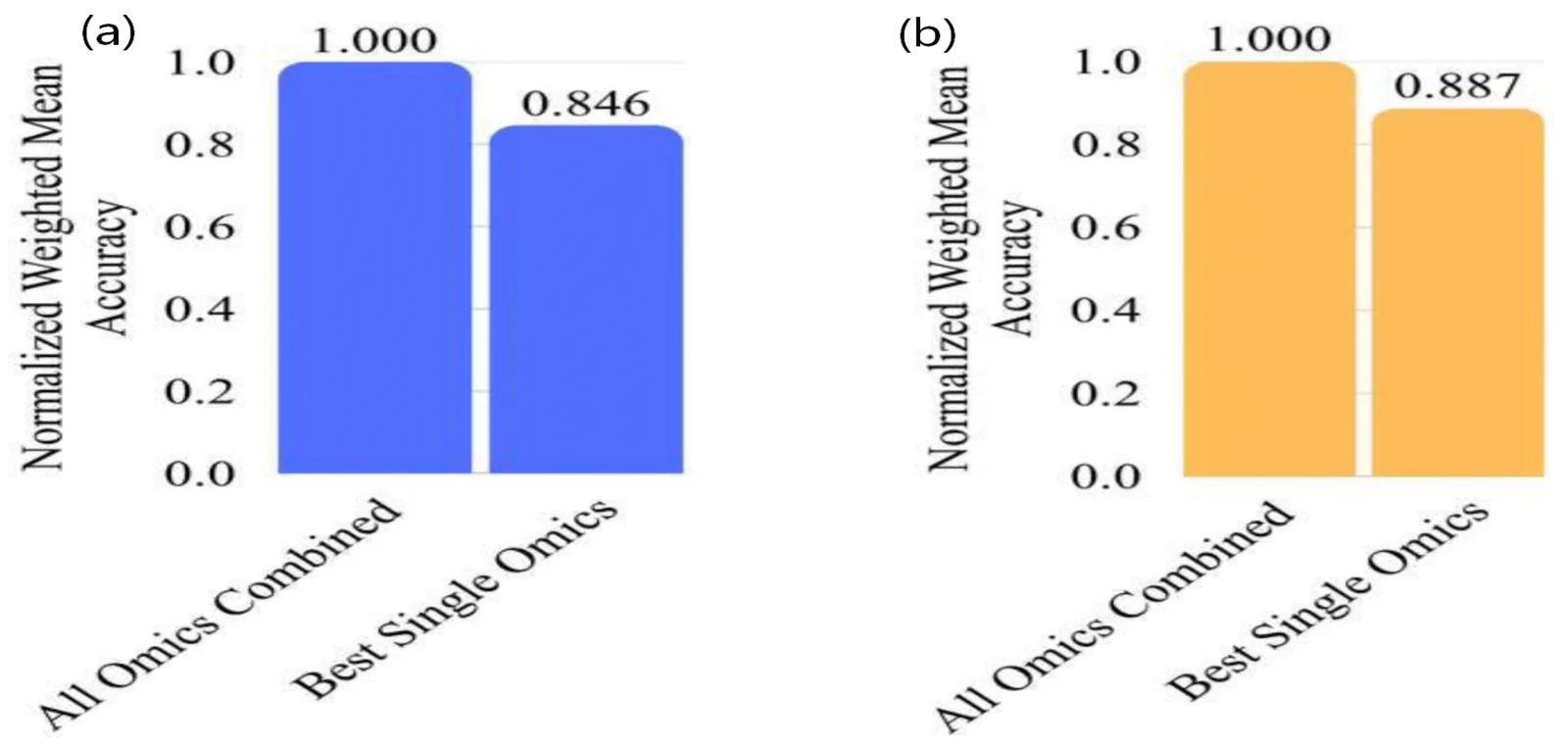
Comparison of best single-omics and integrated multi-omics performance using normalized Weighted Mean Accuracy (NWMA). Comparison of NWMA between integrated all-omics data and the best-performing single-omics feature across the primary and validation datasets, where transcriptomic was identified as the best-performing single-omics feature. (**a**) Blue bars represent NWMA values calculated using results from the primary dataset reported in our previous work [15]. (**b**) Orange bars represent NWMA values calculated from the results in Table 2 Phase 1 of the validation dataset.

This trend is evident in Table 2 Phase 1 (Figure 5b) and in the comparison between the multi-omics results in Table 1 Phase 3 and Table 2 Phase 3 and the single-omics results in Table 1 Phase 2 and Table 2 Phase 2, respectively.

### 3.3. Transcriptomics Shows the Strongest Performance

**Figure 6:**
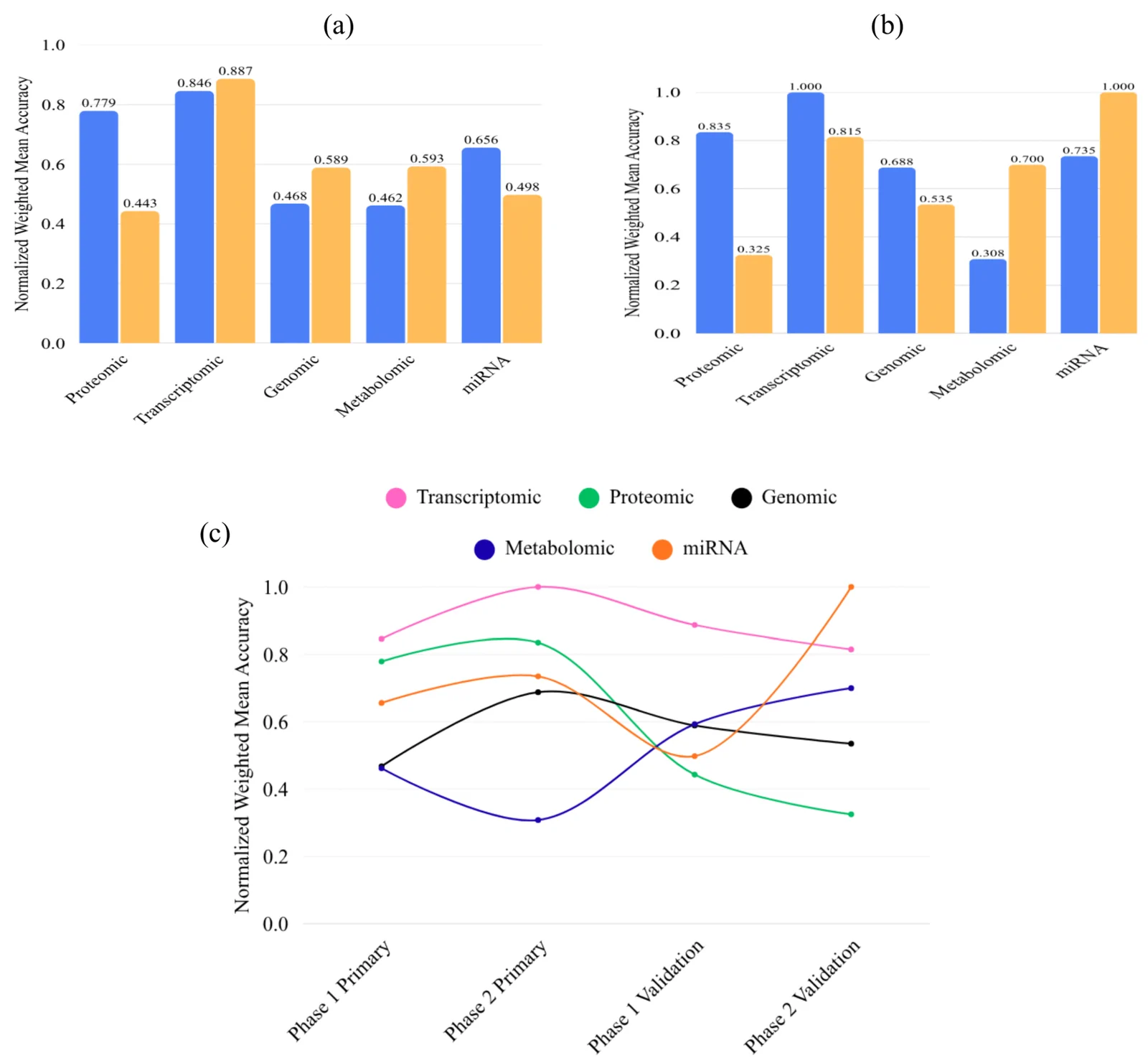
Comparison of normalized Weighted Mean Accuracy (NWMA) across single-omics features for Phase 1 and Phase 2 analyses in the primary and validation datasets. (**a**) NWMA values of different single-omics features calculated using Phase 1 results from the primary dataset reported in our previous work [15] and Phase 1 results from the validation dataset (Table 2). (**b**) NWMA values of different single-omics features calculated using Phase 2 results from Table 1 and Table 2 for the primary and validation datasets, respectively. (**c**) Combined comparison of NWMA trends across Phase 1 and Phase 2 in both the primary and validation datasets for different omics features. In panels (**a**,**b**), blue bars represent NWMA values from the primary dataset, while orange bars represent NWMA values from the validation dataset.

Among single-omics data, transcriptomics demonstrated the best predictive performance, as is evident from Table 2 Phase 1 and Table 1 Phase 2 (Figure 6).

### 3.4. Silhouette Score Influence

**Figure 7:**
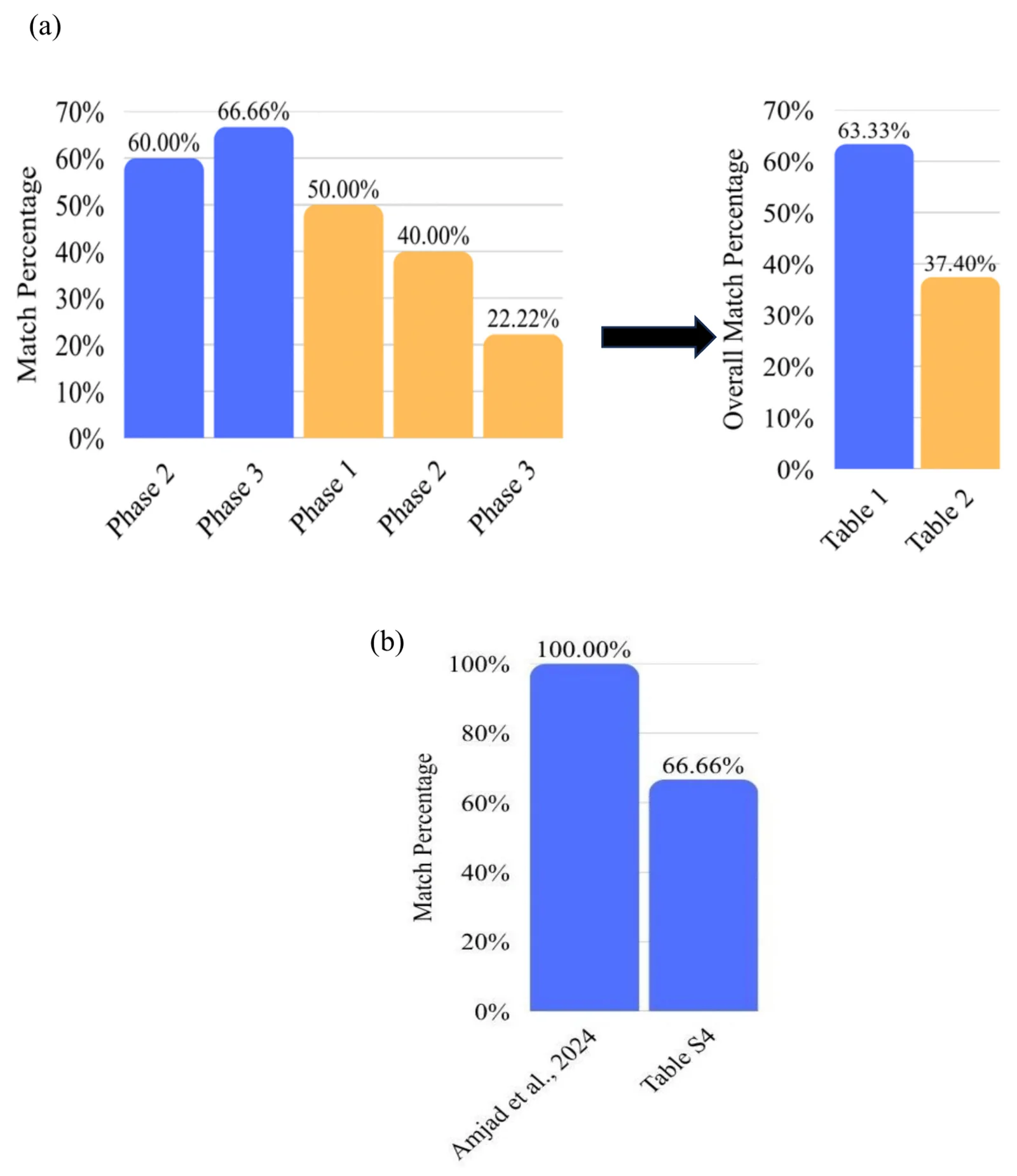
Representation of match percentage across different phases in the primary and validation datasets. (**a**) Match percentage calculated across different phases using results from Table 1 and Table 2, followed by calculation of the overall match percentage for the primary and validation datasets. (**b**) Match percentage calculated using results from Table S4 and the primary dataset results reported in our previous work “Amjad et al., 2024 [15]”. Blue bars represent values from the primary dataset, while orange bars represent values from the validation dataset.

In the primary and validation datasets, the overall match is 63.33% (Table 1) and 37.40% (Table 2), respectively. Additionally, Table S4, containing a comparison of predictive algorithms, showed a 66.66% match (Figure 7). These observations suggest a potential association between clusters with higher silhouette scores and superior DRP; however, the relationship is not consistently observed across datasets and requires further investigation (refer to the Supporting Information section Calculation of Silhouette Score Influence for details).

### 3.5. Proteomic + Transcriptomic + miRNA Shows Stronger Predictive Capability Compared to Other Multi-OMICS Combinations

**Figure 8:**
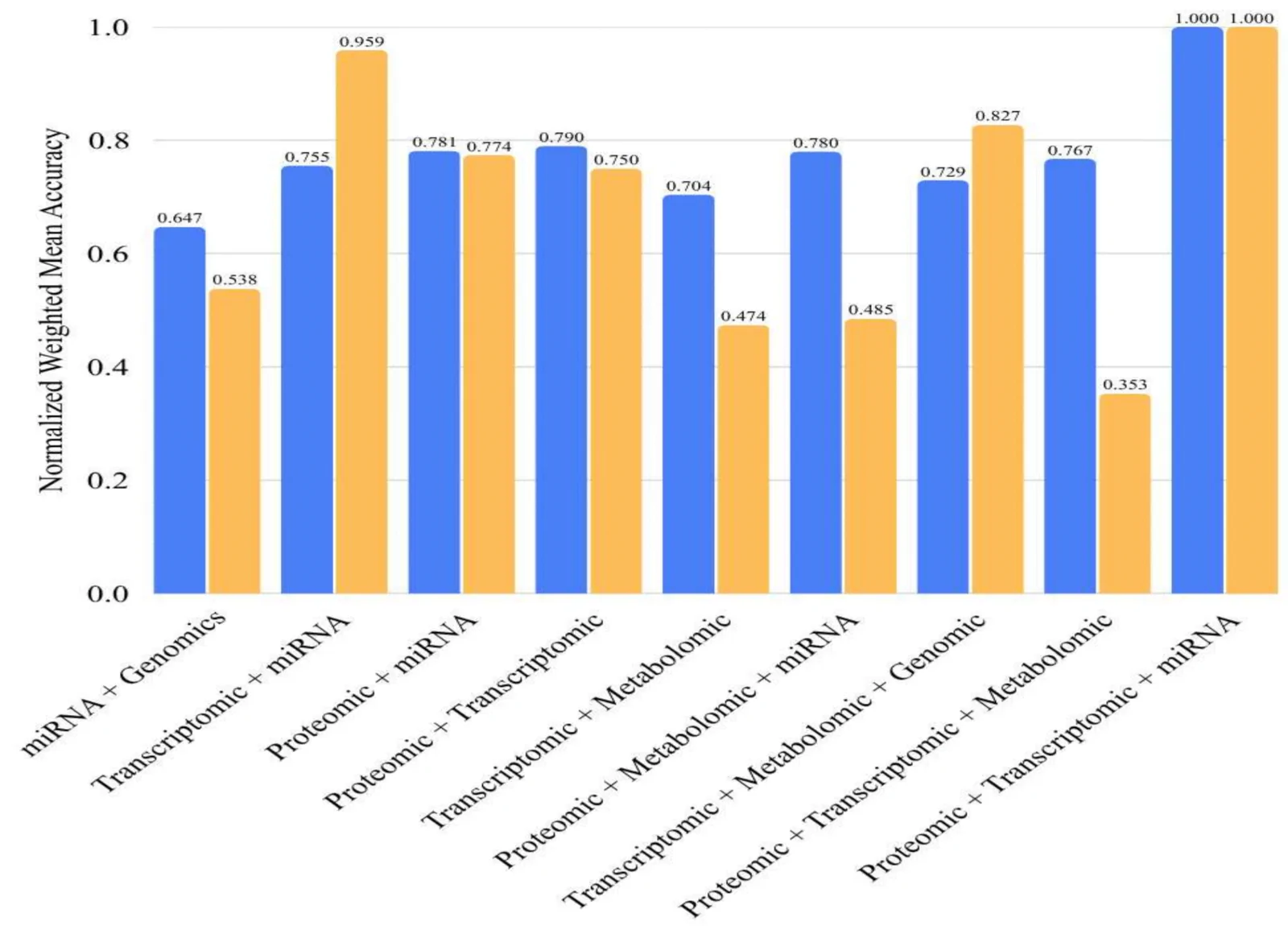
Comparison of normalized Weighted Mean Accuracy (NWMA) across different multi-omics feature combinations in the primary and validation datasets. The figure compares the predictive performance of multiple omics combinations using Phase 3 results from Table 1 and Table 2. Blue bars represent NWMA values from the primary dataset, while orange bars represent NWMA values from the validation dataset.

This trend is visible in Table 1 Phase 3 and Table 2 Phase 3 (Figure 8).

These findings emphasize the value of multi-omics integration and clustering in enhancing drug response prediction (DRP), with the Proteomic + Transcriptomic + miRNA combination demonstrating superior performance, and transcriptomics standing out as the most effective individual feature. The ranking analysis further supports the relative strength and consistency of transcriptomics among single-omics features. These findings also highlight the importance of cluster quality (as reflected by silhouette score) and feature set optimization in improving predictive performance.

## 4. Novel Approach to Drug Response Prediction

To address the lack of a widely accepted standard for identifying the most informative omics feature or feature set for drug response prediction, we propose a framework that identifies the most informative feature and enables systematic ranking of omics layers based on their predictive strength. This study introduces a systematic evaluation framework for quantifying the contribution of multi-omics and single-omics features to drug response prediction. The framework is built on standard classification accuracy and introduces a complementary aggregation metric, Weighted Mean Accuracy (WMA), to assess feature strength by jointly considering predictive performance and the number of drugs with high prediction accuracy.

There are different models and approaches available for enhancing DRP [21,22,23]; however, studies [24] show that there is a need for better predictive models to personalize cancer treatment. In addition to this, we emphasize the need for systematic evaluation of how different omics features contribute to drug response. While extensive work is ongoing in this field, current approaches often fall short in addressing the complexity and variability of treatment responses, particularly in systematically comparing the contribution and consistency of different omics features across datasets.

The proposed approach enables ranking of single-omics features, identification of the optimal multi-omics feature set, and evaluation of the impact of clustering on drug response prediction. The framework also provides a targeted perspective by focusing on drugs with high prediction accuracy, resulting in a more robust and interpretable analysis.

Furthermore, the framework is evaluated using an independent drug screening dataset (PRISM Repurposing Secondary Screen [20]), demonstrating its generalizability and consistency across datasets. This provides a robust and structured strategy for evaluating the utility of omics features in drug response prediction.

## 5. Discussion

This study addresses the challenge of identifying the most informative omics features for drug response prediction across pan-cancer cell lines. Using a systematic evaluation framework, we show that transcriptomic data are the most informative among single-omics features, while the combination of transcriptomic, proteomic, and miRNA features provides the most optimal performance among multi-omics feature sets across both primary and validation datasets.

The integration of multi-omics data for drug sensitivity prediction in cancer cell lines demonstrated improved predictive performance, validating the effectiveness of combining biological features.

Most existing predictive models in the drug discovery and precision oncology domain, including MOLI [21], DeepDR [25], KESVR [23], and network-based pan-cancer approach [26], focus on drug-related prediction tasks. A limited number of studies have focused on identifying informative omics features; for example, Iorio et al. applied Elastic Net and Random Forest models to predict continuous IC_50_ values across pan-cancer cell lines using mutations, copy number alterations, methylation, and gene expression data, with gene expression emerging as one of the most informative molecular data types in their analyses [7]. In comparison, our study focuses on systematically evaluating and ranking the contribution of omics features to drug response prediction, rather than optimizing predictive performance. Consistent with prior findings (e.g., Iorio et al. [7]), our study identifies transcriptomic data as one of the most informative features and further identifies the most informative omics feature set for drug response prediction using pan-cancer omics data, including transcriptomic, proteomic, metabolomic, miRNA, and genomic data. Therefore, the proposed framework should be viewed as complementary to existing predictive models, focusing on feature evaluation rather than prediction optimization.

In this study, t-SNE is used for dimensionality reduction. As previous studies across diverse domains have demonstrated the utility of t-SNE embeddings for predictive tasks [27,28,29], its systematic use as direct input features within a cluster-centric framework for omics feature strength evaluation remains underexplored. Here, t-SNE is not used for visualization; instead, the resulting low-dimensional embeddings are directly employed as input features for clustering and downstream drug response prediction. t-SNE ensures that points (cell lines) that are close in the high-dimensional space remain close in the low-dimensional space. However, it does not preserve global structure, meaning that the relative distances between distinct groups of similar cell lines may not be accurately maintained. To address this, we focused on the preserved local structure by performing clustering on the t-SNE-reduced features and evaluated the quality of the clusters using the silhouette score. This approach demonstrates that the clustering effectively captures local similarities between cell lines, aligning with the objective of examining the relationship between cluster quality and predictive performance.

**Figure 9:**
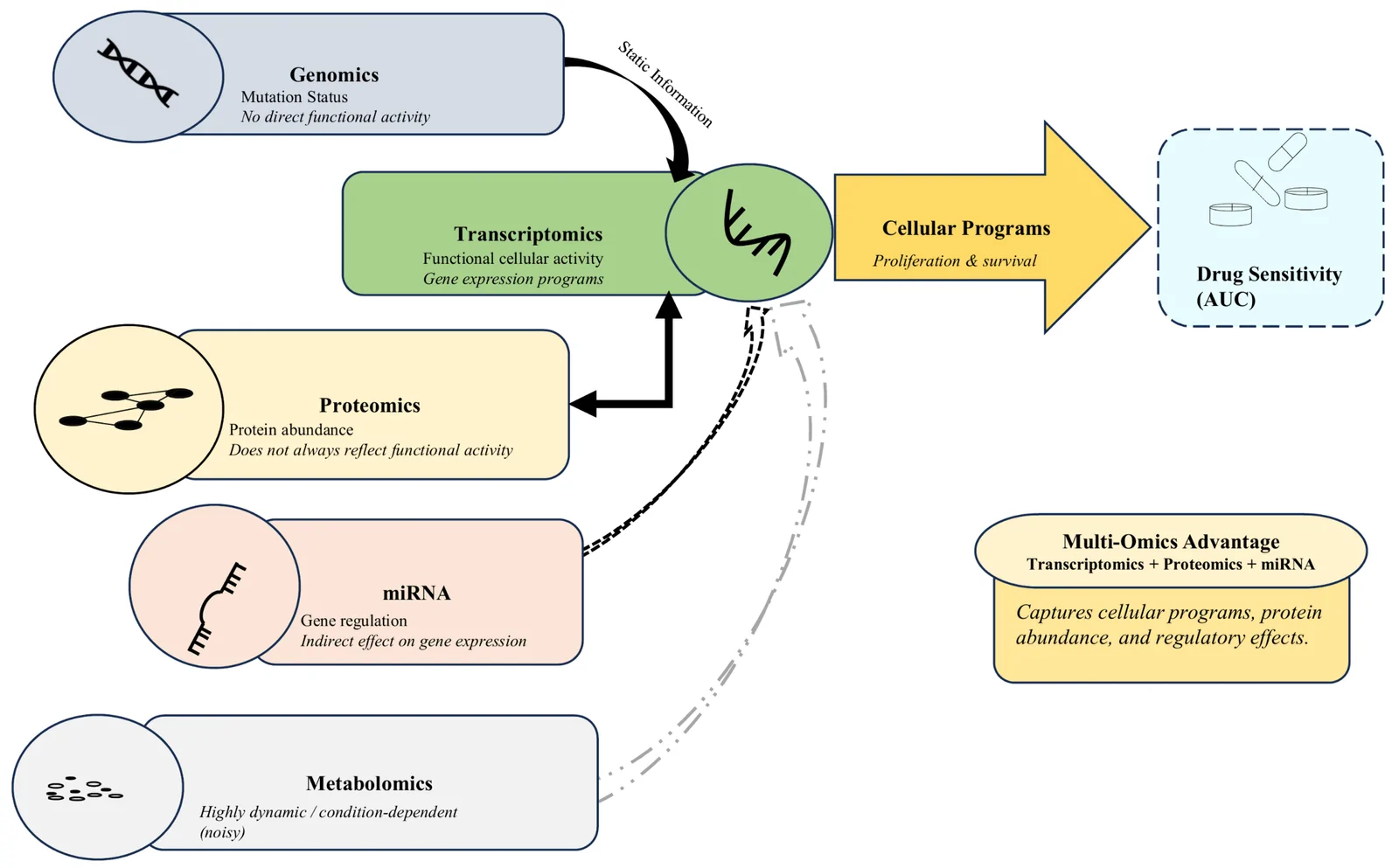
Biological interpretation of omics contribution to drug sensitivity. Illustration of how different omics layers contribute to cellular programs and drug sensitivity. Solid arrows indicate molecular relationships between omics layers, dashed arrows indicate indirect regulatory relationships, and light gray dash-dotted arrows indicate dynamic or condition-dependent relationships. Bidirectional arrows indicate interconnected relationships between molecular layers.

Our results consistently show that multi-omics combinations show stronger predictive capability than single-omics features in predicting drug responses. Specifically, the combination of transcriptomic, proteomic, and miRNA data was found to be the most effective in our study, as it yielded the best observed performance across both primary and validation datasets. This finding is also supported by our previous work [30], where we performed 5-fold cross-validation using RFC. This indicates that the integration of transcriptomics, proteomics, and miRNA captures a broader spectrum of biological signals, leading to improved DRP. Interestingly, in this study, transcriptomic data alone exhibited strong predictive performance, surpassing other single-omics features. Prior large-scale evaluations across 33 TCGA cancer types using penalized Cox models have also shown that gene expression data outperforms other omics (e.g., copy number variation and somatic point mutation) in predictive performance when used for predicting patient survival outcomes [31]. In a study by Iorio et al., among the molecular data types considered (mutations, copy number alterations, methylation, and gene expression), gene expression was identified as the most informative feature for predicting drug response [7]. This highlights the robustness of transcriptomics as a key predictor of drug sensitivity. The finding aligns with an existing study that highlights the utility of transcriptome profiling in cancer diagnosis and therapy guidance [32]. Moreover, transcriptomics reflects cellular programs associated with cancer cell growth and cell death. Because drug AUC measures drug sensitivity based on cell viability, transcriptomic data directly relates to AUC by capturing the cellular programs that control proliferation and survival. Transcriptomics serves as a bridge between the static genome and the functional proteome. Proteomics can be used for predicting drug sensitivity; however, protein abundance alone does not always indicate whether a protein is functionally active, so measuring protein abundance alone may lead to weaker predictions. miRNA affects drug response indirectly by fine-tuning gene expression, which is why it contributes modestly but is not strongly predictive on its own. Metabolomic data are often noisy and unstable because metabolite levels change rapidly with nutrient availability, culture conditions, and cellular stress. Genomic data are discrete and indicate whether specific mutations are present or absent, but they do not capture pathway activity or functional cellular state, making them the most indirect with respect to drug sensitivity magnitude. Transcriptomics, proteomics, and miRNA together describe cell behavior more completely compared to single-omics features, so they predict drug sensitivity better, since they reflect what the cell is doing, what proteins it has, and how strongly these processes are regulated (refer to Figure 9).

In our previous work, Table 1 [15] demonstrated a 100% match between the cluster with the highest silhouette score and the cluster performing best for DRP, suggesting a strong correlation. Furthermore, comparing the predictive performance obtained using the best cluster with a silhouette score (~0.4) in the primary dataset (Table 1) [15] to that with silhouette scores (~0.2) in the validation dataset (Table 2 Phase 1) revealed that the primary dataset consistently produced better outcomes. These findings suggest that while the silhouette score is a critical factor, predictive performance may also be influenced by other biological characteristics, which warrant further investigation. Further analysis of Table 1 [15] and Table 2 Phase 1 reveals that both tables show better results for clustering compared to without clustering. In Table 1 [15], transcriptomics achieves the highest performance among single-omics, while miRNA shows comparatively lower performance. These observations underscore that the consistent performance of transcriptomics makes it the most informative single-omics feature for DRP. Furthermore, the analysis of the tables generated from the primary dataset, specifically Table 1 [15] and Table 1 Phase 2, indicates that the performance of proteomics is comparable to that of transcriptomics. However, upon examining the validation dataset results shown in Table 2 Phase 1 and Table 2 Phase 2, it is evident that transcriptomics demonstrates greater consistency and reliability (refer to Supporting Information for an extended discussion).

## 6. Conclusions

In conclusion, this study identifies the most informative feature set within the evaluated datasets, consisting of proteomic, transcriptomic, and miRNA features, which yielded the highest observed performance, highlighting the utility of the multi-omics approach. Among the standalone omics features, transcriptomics consistently performed well, demonstrating its strength in drug sensitivity prediction. This research supports the value of integrating diverse biological features to improve drug sensitivity predictions, thereby opening opportunities for more personalized therapeutic strategies in cancer treatment.

### Future Work and Limitations

This study has certain limitations. The relationship between silhouette score and predictive performance remains unclear. The use of clustering criteria (such as minimum cluster size and silhouette threshold) also limited the inclusion of certain omics combinations, indicating the need for improved cluster-handling strategies. Exploring dimensionality-reduction methods beyond PCA and t-SNE may further improve the framework. Incorporating additional omics layers may provide a broader assessment of their contribution to drug response prediction.

Future research should focus on applying the proposed framework to larger and more diverse datasets to improve robustness and generalizability. Further validation using patient-derived and clinical multi-omics datasets is needed to assess the clinical applicability of the proposed framework. Moreover, the observed prioritization of omics layers is specific to cancer drug response, and further studies are needed to determine whether this prioritization applies to other biological or disease contexts.

## Figures and Tables

**Table 1:** Primary dataset results for Phase 2 and Phase 3.

Primary Dataset Results						
Phase 2						
Omics Datatype		Cluster 0	Cluster 1	Cluster 2	Cluster 3	Cluster 4
Proteomic	Mean Accuracy	0.80	0.90	0.90	0.82	0.84
	Number of drugs with accuracy more than75%	44	16	12	21	27
Transcriptomic	Mean Accuracy	0.82	0.80	0.85	0.86	0.82
	Number of drugs with accuracy more than75%	42	22	33	15	33
Genomic	Mean Accuracy	0.81	0.83	0.82	0.83	0.84
	Number of drugs with accuracy more than75%	17	19	23	29	12
Metabolomic	Mean Accuracy	0	0.79	0.77	0.80	0.82
	Number of drugs with accuracy more than75%	0	10	6	7	23
miRNA	Mean Accuracy	0.80	0.83	0.83	0.84	0.92
	Number of drugs with accuracy more than75%	33	28	23	9	13
Phase 3						
miRNA + Genomic	Mean Accuracy	0.82	0.81	0.82	0.80	0.87
	Number of drugs with accuracy more than 75%	28	11	23	21	21
Transcriptomic + miRNA	Mean Accuracy	0.82	0.87	0.80	0.86	0.84
	Number of drugs with accuracy more than 75%	43	5	14	32	26
Proteomic + miRNA	Mean Accuracy	0.81	0.80	0.83	0.87	0.84
	Number of drugs with accuracy more than 75%	40	11	20	14	40
Proteomic + Transcriptomic	Mean Accuracy	0.84	0.84	0.85	0.82	0.81
	Number of drugs with accuracy more than 75%	42	19	13	33	19
Transcriptomic + Metabolomic	Mean Accuracy	0.82	0.81	0.85	0.83	0.85
	Number of drugs with accuracy more than 75%	42	30	15	13	13
Proteomic + Metabolomic +miRNA	Mean Accuracy	0.83	0.82	0.83	0.84	0.87
	Number of drugs with accuracy more than 75%	36	19	27	31	11
Transcriptomic + Metabolomic + Genomic	Mean Accuracy	0.80	0.82	0.85	0.83	0.83
	Number of drugs with accuracy more than 75%	46	24	21	16	11
Proteomic + Transcriptomic + Metabolomic	Mean Accuracy	0.81	0.85	0.81	0.83	0.84
	Number of drugs with accuracy more than 75%	40	23	10	32	18
Proteomic + Transcriptomic + miRNA	Mean Accuracy	0.84	0.90	0.84	0.82	0.82
	Number of drugs with accuracy more than 75%	44	25	49	12	27

Represents results from the primary dataset for Phase 2 and Phase 3 of the implementation process. Mean accuracy is the mean of the prediction accuracy of drugs with accuracy above 75%. The green-colored cells represent executions in which the highest silhouette score cluster yielded the best results for drug response prediction. The match percentage was calculated by dividing the number of rows with green-colored cells by the total number of rows and multiplying by 100 in each phase. The overall match percentage is the mean of the match percentages across all phases. The results of Phase 1 are presented in our previous paper (Table 1) [15].

**Table 2:** Validation dataset results for Phase 1, Phase 2, and Phase 3.

Validation Dataset Results					
Phase 1					
Omics Datatype		Cluster 0	Cluster 1	Cluster 2	Without Clustering
All Omics	Mean Accuracy	0.80	0.78	0.80	0.77
	Number of drugs with accuracy more than 75%	9	4	5	2
Proteomic	Mean Accuracy	0.78	0.76	0.81	0.76
	Number of drugs with accuracy more than 75%	4	2	2	1
Transcriptomic	Mean Accuracy	0.78	0.79	0.77	0.78
	Number of drugs with accuracy more than 75%	10	5	2	1
Genomic	Mean Accuracy	0.78	0.77	0.79	0
	Number of drugs with accuracy more than 75%	4	5	3	0
Metabolomic	Mean Accuracy	0.78	0.82	0.78	0.78
	Number of drugs with accuracy more than 75%	5	1	5	1
miRNA	Mean Accuracy	0.79	0.79	0.79	0
	Number of drugs with accuracy more than 75%	3	3	4	0
Phase 2					
Proteomic	Mean Accuracy	0.80	0.76	0.78	
	Number of drugs with accuracy more than75%	2	1	5	
Transcriptomic	Mean Accuracy	0.78	0.79	0.77	
	Number of drugs with accuracy more than75%	6	13	1	
Genomic	Mean Accuracy	0.79	0.79	0.80	
	Number of drugs with accuracy more than75%	6	3	4	
Metabolomic	Mean Accuracy	0	0.79	0.80	
	Number of drugs with accuracy more than75%	0	10	7	
miRNA	Mean Accuracy	0.77	0.77	0.81	
	Number of drugs with accuracy more than75%	1	3	20	
Phase 3					
miRNA + Genomic	Mean Accuracy	0.79	0.78	0.78	
	Number of drugs with accuracy more than 75%	7	5	5	
Transcriptomic + miRNA	Mean Accuracy	0.80	0.78	0.79	
	Number of drugs with accuracy more than 75%	8	3	19	
Proteomic + miRNA	Mean Accuracy	0.79	0.80	0.80	
	Number of drugs with accuracy more than 75%	4	4	16	
Proteomic + Transcriptomic	Mean Accuracy	0.81	0.76	0.81	
	Number of drugs with accuracy more than 75%	11	1	11	
Transcriptomic + Metabolomic	Mean Accuracy	0.77	0.78	0.79	
	Number of drugs with accuracy more than 75%	2	6	7	
Proteomic + Metabolomic +miRNA	Mean Accuracy	0.78	0.80	0.81	
	Number of drugs with accuracy more than 75%	3	6	6	
Transcriptomic + Metabolomic + Genomic	Mean Accuracy	0.79	0.77	0.80	
	Number of drugs with accuracy more than 75%	5	9	12	
Proteomic + Transcriptomic + Metabolomic	Mean Accuracy	0.78	0.80	0.79	
	Number of drugs with accuracy more than 75%	2	6	3	
Proteomic + Transcriptomic + miRNA	Mean Accuracy	0.79	0.80	0.80	
	Number of drugs with accuracy more than 75%	4	5	22	

Represents results from the validation dataset for Phase 1, Phase 2, and Phase 3 of the implementation process. Mean accuracy is the mean of the prediction accuracy of drugs with accuracy above 75%. The green-colored cells represent executions in which the highest silhouette score cluster yielded the best results for drug response prediction. The match percentage was calculated by dividing the number of rows with green-colored cells by the total number of rows and multiplying by 100 in each phase. The overall match percentage is the mean of the match percentages across all phases.

**Table 3:** Rank analysis.

Scenario 1: Rank Difference and Sum for Figure 3a				
**Omics Feature**	Rank in Primary	Rank in Validation	Rank Difference D_i_	Rank Sum S_i_
**All Omics**	1	1	0	2
**Transcriptomic**	2	2	0	4
**Proteomic**	3	6	3	9
**miRNA**	4	5	1	9
**Genomic**	5	4	1	9
**Metabolomic**	6	3	3	9
Scenario 2: Rank Difference and Sum for Figure 3b				
**Transcriptomic**	1	2	1	3
**Proteomic**	2	5	3	7
**miRNA**	3	1	2	4
**Genomic**	4	4	0	8
**Metabolomic**	5	3	2	8
Total Rank Difference and Sum across both Scenarios				
	Total Rank Difference (D_i_ + D_i_)		Total Rank Sum (S_i_ + S_i_)	
**All Omics**	0 (Only in Scenario 1)		2(Only in Scenario 1)	
**Transcriptomic**	0 + 1 = 1		4 + 3 = 7	
**Proteomic**	3 + 3 = 6		9 + 7 = 16	
**miRNA**	1 + 2 = 3		9 + 4 = 13	
**Genomic**	1 + 0 = 1		9 + 8 = 17	
**Metabolomic**	3+ 2 = 5		9 + 8 = 17	

Represents Rank Difference (D_i_) and Rank Sum (S_i_) calculated using Figure 3a,b. D_i_ is the difference between the primary and validation dataset ranks of omics feature I, and Si is the sum of the primary and validation dataset ranks of omics feature i. Table 3 Scenario 1 is generated using Figure 3a, and Table 3 Scenario 2 is generated using Figure 3b. Total Rank Difference is the sum of Rank Difference (D_i_) calculated in Scenario 1 and Scenario 2 for omics feature i. Total Rank Sum is the sum of Rank Sum (S_i_) calculated in Scenario 1 and Scenario 2 for omics feature i.

## Data Availability

The original data presented in the study are openly available in the DepMap portal at https://depmap.org/portal/ (accessed on 27 May 2025), and the original contributions presented in this study are included in the article/Supplementary Material. Further inquiries can be directed to the corresponding authors.

## Supplementary Materials

The following supporting information can be downloaded at: https://www.mdpi.com/article/10.3390/cimb48090897/s1.
