# Supplementary material for: A Systematic Machine Learning Framework for Evaluating and Ranking Omics Layers in Cancer Drug Response Prediction
Source: Curr Issues Mol Biol. 2026 Sep 2;48(9):897. doi: 10.3390/cimb48090897 (PMC13606002; doi:10.3390/cimb48090897)
Supplement: Supplementary file 1 [file cimb-48-00897-s001.zip › Supporting Information.pdf]

Supporting Information

Extended Preprocessing

To manage high dimensionality and retain essential molecular characteristics, we applied scaling to standardize feature contribution, followed by t-SNE (t-distributed Stochastic Neighbor Embedding) for dimensionality reduction on each individual omics dataset. t-SNE effectively reduced the feature space while retaining key patterns relevant to our analysis. Following dimensionality reduction, the transformed features were stored in a MySQL database to facilitate easier data management. After completing the dimensionality reduction for each omics dataset, we integrated the reduced feature sets into a single multi-omics dataset. The integration process involved joining the t-SNE-reduced features together as well as with drug sensitivity data (AUC values) via SQL queries. This ensured that the multi-omics data were aligned with the drug response profiles of the cell lines. It resulted in an integrated multi-omics dataset comprising 16 features, which was subsequently used as input for the clustering algorithm for further analysis [15].

Choice of K-Means for Clustering

In the primary dataset, among the various algorithms evaluated, K-Means exhibited a silhouette score of 0.373375 for Cluster 0, with the k value set to 5, reflecting its effectiveness in achieving clear separation between clusters. In the Table S2, K-Means achieved an overall silhouette score of 0.170057, which was better than all other clustering algorithms (ignoring K-Means++, as it performed the same as K-Means) and a Davies–Bouldin Index value of 1.854821. This trend indicates that K-Means maintains better-defined clusters overall. The K-Means overall silhouette score of 0.170057 in the Table **S2** was better than all other clustering algorithms, such as Agglomerative Clustering (0.146592) and Gaussian Mixture Model (GMM) (0.146520). Spectral Clustering, despite producing decent separation for Cluster 1 (0.377002), resulted in a lower overall silhouette score of 0.160152, highlighting potential issues with cluster stability across different subsets of data. Agglomerative Clustering performed relatively well for Cluster 3 (0.382595) but had an overall silhouette score of only 0.146592, suggesting that its hierarchical approach may not be effective in this context. Additionally, K-Means outperformed other algorithms in terms of the Davies–Bouldin Index, recording a value of 1.854821, except for Mini-Batch K-Means (1.823567). This index measures the average similarity ratio of each cluster with the one most similar to it, with lower values indicating better clustering. Mini-Batch K-Means, which is designed to handle larger datasets by using smaller subsets of data, achieved a lower overall silhouette score of 0.169679, as well as a lower silhouette score for the best-performing cluster when compared to K-Means. Thus, K-Means was chosen after analyzing three key criteria: individual cluster silhouette score, overall silhouette score, and Davies–Bouldin Index. Moreover, K-Means is easy to implement and interpret, making it well-suited for the feature strength evaluation framework proposed in this study.

Experimenting with k Value

We experimented with several values of k (the number of clusters), ranging from 2 to 9 (Table S3), and calculated the silhouette score for each configuration to assess cluster quality. The value of k was chosen by focusing on highest silhouette score cluster, to study the impact of a well-defined cluster on drug sensitivity prediction. Cluster 0 repeatedly stood out as having the highest silhouette score, with a maximum score of 0.373375 at k=5. This suggested that Cluster 0 exhibited relatively well-defined boundaries, with clear separation from other clusters. As such, we selected k=5 as the optimal number of clusters and divided the dataset into five separate clusters (Cluster 0, 1, 2, 3, and 4). While Cluster 0 demonstrated good intra-cluster consistency, Clusters 1 to 4 had lower silhouette scores, indicating less distinct boundaries. These clusters, obtained with k = 5, were used to study drug response prediction by treating each cluster as a separate dataset input to the prediction algorithm. Through this approach, we were able to study the relationship between silhouette scores and the framework's predictive performance. Simultaneously, we evaluated the predictive strength of different omics features.

In subsequent sections of this paper, we explore efforts to improve cluster quality and enhance prediction results by experimenting with various combinations of omics features and simultaneously identifying the most informative omics feature and feature combinations.

Selection of Random Forest

The overall mean accuracy of RFC ranged from 0.81 to 0.85 (Table S4), demonstrating particularly strong predictive capabilities in Cluster 0 (0.83), Cluster 3 (0.83) and Cluster 4 (0.85). Furthermore, RFC effectively identified the highest number of drugs achieving an accuracy above 75%, with 39 drugs in Cluster 0, 28 drugs in Cluster 1, and 30 drugs in Cluster 2. A higher number of accurately predicted drugs indicates that the framework performs well across many drugs, reflecting its strength and generalizability. In contrast, other classifiers, such as SVC and AdaBoost Classifier, yielded lower overall mean accuracies and fewer drugs surpassing the 75% accuracy threshold across the clusters. This observed trade-off between mean accuracy and the number of drugs predicted illustrates the rationale for favoring RFC in this analysis.

Implementation of Random Forest Algorithm

(i) During preprocessing, missing AUC values were temporarily encoded as zero to facilitate handling. Prior to training the Random Forest models, these placeholder entries were excluded by removing cell lines with zero AUC values for the specific target. This step ensured that incomplete data did not bias the training process. (ii) Cell lines with AUC values greater than or equal to the mean for a given drug were classified as class 1, while those with AUC values less than the mean were classified as class 0. (iii) To evaluate the performance of the Random Forest models, we employed an 80:20 train-test split, with 80% of the data used for training and 20% reserved for testing. The Random Forest drug response prediction algorithm used in this framework is designed in a way that, in each iteration, a single drug is set as the target variable and the prediction accuracy of the target variable is calculated. The number of

iterations equals the number of target variables (drugs) in the dataset. This not only makes prediction independent of the number of drugs in the dataset but also helps in handling missing values at the time of execution for each drug separately. Thus, the difference in the number of drugs in the primary and validation datasets doesn't impact the prediction results.

Model accuracies were calculated by comparing the predicted drug sensitivity labels with the actual labels from the test set. The accuracy for each target variable (drug) was calculated as the ratio of the number of correct predictions to the total number of test records. Mean accuracy is the mean of the prediction accuracy of drugs with accuracy above 75%.

Extended Multi-Omics Selection

In the primary dataset (Table 1 Phase 3), the transcriptomic + proteomic + miRNA combination achieves high mean accuracies, peaking at 0.90 in Cluster 1 and maintaining competitive values across other clusters (0.82 to 0.90). It also predicts a significant number of drugs with accuracy over 75%, especially in Cluster 2, which has 49 drugs, and Cluster 0 with 44 drugs. In the validation dataset (Table 2 Phase 3), this combination maintains strong mean accuracies around 0.80 and stands out in Cluster 2 with 22 drugs predicted above 75% accuracy, outperforming most other combinations. This consistent performance across datasets suggests that integrating transcriptomic, proteomic, and miRNA data captures a broader range of biological signals, leading to better DRP.

Weighted Mean Accuracy (WMA)

Example Calculation for Transcriptomic (Table 2 Phase 1)

$$\text{WMA}_{\text{Transcriptomic}} = (0.78 \times 10) + (0.79 \times 5) + (0.77 \times 2) + (0.78 \times 1)$$
$$= 14.07$$

Similarly, for other omics in Table 2 Phase 1

Here are the calculated Weighted Mean Accuracy (WMA) values for each omics type:

$\text{WMA}_{\text{Combined Omics}}$: 15.86

$\text{WMA}_{\text{Proteomic}}$: 7.02

$\text{WMA}_{\text{Transcriptomic}}$: 14.07

$\text{WMA}_{\text{Genomic}}$: 9.34

$\text{WMA}_{\text{Metabolomic}}$: 9.4

$\text{WMA}_{\text{miRNA}}$: 7.9

$\text{WMA}_{\text{Combined Omics}} = \text{Max (WMA)}$

Max (WMA) = 15.86

1. **Normalization of WMA**

Example Calculation for Transcriptomic (Table 2 Phase 1)

$$\text{NWMA}_{\text{Transcriptomic}} = \frac{14.07}{15.86} = 0.8871$$

NWMA values for other feature sets in Table 2 Phase 1

$\text{NWMA}_{\text{Combined Omics}}$: 1.0000

$\text{NWMA}_{\text{Proteomic}}$: 0.4426

$\text{NWMA}_{\text{Transcriptomic}}$: 0.8871

$\text{NWMA}_{\text{Genomic}}$: 0.5889

$\text{NWMA}_{\text{Metabolomic}}$: 0.5927

$\text{NWMA}_{\text{miRNA}}$: 0.4981

Calculation of Silhouette Score Influence

In the primary dataset used for the current study, Table 1 Phase 2 showed a 60% match, and Table 1 Phase 3 showed a 66.66% match, resulting in an overall match of 63.33%. These results indicate that clusters with higher silhouette scores frequently exhibit superior predictive performance, reinforcing the idea that well-defined clusters are more biologically informative for drug response prediction. For the validation dataset, Table 2 Phase 1 showed a 50% match, Table 2 Phase 2 showed a 40% match, and Table 2 Phase 3 showed a 22.22% match, culminating in an overall match of 37%. While we do accept that these results are less robust than those observed with the primary dataset, they still point to a potential relationship between high silhouette scores and predictive performance, albeit with reduced strength. The match percentage was calculated by dividing the number of rows with green-colored cells by the total number of rows and multiplying by 100.

Extended Discussion

In the primary dataset, higher silhouette scores were strongly associated with better drug response predictions, underscoring the critical role of cluster quality in reliable results. However, this trend was less pronounced in the validation dataset, suggesting that other biological factors also play a crucial role in drug response prediction. To prevent data leakage, we ensured that t-SNE dimensionality reduction was performed using only the omics feature set, with the target variable (AUC values) incorporated exclusively during the training and testing phases of the predictive analysis. This careful preprocessing ensured that the target variable did not influence either the dimensionality reduction or clustering steps, thereby enhancing the robustness and reliability of our analysis.

We have shown the high accuracy of drugs identified using the same framework in our previous paper [15] as bar graphs to provide a clear overview of the framework's performance across different clusters. The results presented in Table 2 Phase 1 in the current study are also supported by our previous findings, shown in Table 1 [15], where similar trends were observed: Clustering improves performance, multi-

omics outperforms single-omics and transcriptomics excels. The comparison of single-omics and multi-omics is represented in Fig 3a. In our analysis, we removed a significant number of records due to missing values after performing dimensionality reduction. Despite this, the reduced features remain informative and relevant for drug response prediction, as evidenced by consistent results across the primary and validation datasets as well as the biological significance of the results. Dimensionality reduction was performed separately for each omics feature, ensuring that the local structure within each dataset was maintained. Since the primary goal was to identify the most relevant omics features for predicting drug response, this approach achieves its objective, with the remaining records preserving sufficient meaningful relationships in the dataset. In the single-omics setting (specifically in Table 3 Scenario 2), each omics type is evaluated independently, eliminating any potential bias introduced by unequal feature counts. The rank-based analysis reveals that genomic and metabolomic features perform the worst when considered individually. This aligns with the results observed in the multi-omics integration, where the optimal feature set excludes genomic and metabolomic data. Thus, the consistency between single-omics and multi-omics results confirms that the theoretical imbalance in feature counts after t-SNE reduction does not introduce significant bias in the framework's outcome. Density-based clustering, such as DBSCAN was not included in this analysis, as we did not want to compromise data by treating potentially informative samples as noise. This study used K-Means with a fixed number of clusters across different omics combinations, allowing for consistent comparison of predictive performance across clusters. However, DBSCAN, being a density-based clustering method, determines the number of clusters based on chosen parameters and data density. As a result, applying DBSCAN to different omics combinations could produce a varying number of clusters, making direct performance comparison between combinations more complex and less controlled.

Algorithm

Algorithm 1: Preprocessing of Omics Datasets

Step 1: Download each Omics Feature file separately from DepMap Portal.

Step 2: Perform Dimensionality Reduction Using t-SNE.

For each omics dataset:

– Apply StandardScaler() and TSNE (n_components=X, random_state=42) on single-omics feature set. [Note: X is adjusted based on empirical results]

– Store the t-SNE output as a reduced omics table.

– Repeat for all individual omics datasets.

Step 3: Integrate Single-Omics Datasets Using SQL Join.

– Use SQL INNER JOIN across all omics tables using cell_line as the common key.

– Merge the resulting omics data with drug sensitivity data (AUC values) .

– This join operation generates final dataset that consists of overlapping cell_line in all the tables.

Algorithm 2: Stepwise Representation of the Novel Cluster-Based Drug Response Prediction Framework

Step 1: Import Required Python Libraries

– Load libraries: Pandas, NumPy, Scikit-learn (KMeans, RandomForestClassifier, metrics), Matplotlib, SQLAlchemy

Step 2: Establish MySQL Database Connection

– Define database credentials
– Connect to the MySQL server
– Load integrated multi-omics dataset.

Step 3: Define Omics-Based Feature Sets for Clustering

– miRNA: mirna_TSNE1, mirna_TSNE2
– Metabolomics: metabolomics_TSNE1, metabolomics_TSNE2
– CNA: Dimension_1_cna, Dimension_2_cna, Dimension_3_cna
– Expression: Dimension_1_expression, Dimension_2_expression, Dimension_3_expression
– Mutation: Dimension_1_mutation, Dimension_2_mutation, Dimension_3_mutation
– Proteomics: Dimension_1_protein_array, Dimension_2_protein_array, Dimension_3_protein_array

Step 4: Apply K-Means Clustering by giving feature set as an input to K-Means Clustering algorithm

– Manually change value of k form k = 2 to 9
– For each value of k:
  - Apply k-means clustering to the selected omics features
  - Compute Silhouette Score and Davies-Bouldin Index
– Select optimal k values:
  - Primary dataset: k = 5
  - Validation dataset: k = 3
– Assign cluster labels to each cell line
– Save each cluster subset as a **separate table** in the MySQL database

Step 5: Evaluate Cluster Quality

– Compute the number of samples in each cluster
– Calculate silhouette score for each cluster to assess internal cohesion

Step 6: Define Drug Targets for Classification

– Use drug AUC values as target variables for classification tasks

Step 7: Apply Random Forest Classification per Cluster

For each cluster:
– Retrieve data from the corresponding cluster-specific table
– For each drug:
  - Exclude rows with zero AUC values
  - Convert continuous AUC values into binary classes [0 if AUC < drug.mean() vs. 1 if AUC ≥ drug.mean()]
  - **Define the classifier**: RandomForestClassifier(n_estimators=300, random_state=42)
  - Split the data: 80% training, 20% testing (random_state=42)
  - Train the model using training data
  - Evaluate model performance using accuracy score
  - If accuracy > 75%, store the drug as highly predictable

Step 8: Visualization

– Generate accuracy bar plots per drug within each cluster
– Highlight drugs achieving prediction accuracy > 75%
– Count the number of drugs with accuracy > 75% and calculate Mean accuracy of drugs with accuracy>75%

Step 9: Display Results

– Print the list of highly predictable drugs (accuracy > 75%) per cluster
