# Supplementary material for: A Systematic Machine Learning Framework for Evaluating and Ranking Omics Layers in Cancer Drug Response Prediction
Source: Curr Issues Mol Biol. 2026 Sep 2;48(9):897. doi: 10.3390/cimb48090897 (PMC13606002; doi:10.3390/cimb48090897)
Supplement: Supplementary file 1 [file cimb-48-00897-s001.zip › Table S1.pdf]

Table S1. Silhouette Score for PCA and t-SNE.

Omics Type	Method	Average Silhouette Score
miRNA	PCA	0.1392
miRNA	t-SNE	0.4767
Metabolomic	PCA	0.1205
Metabolomic	t-SNE	0.4533
