# Supplementary material for: A Systematic Machine Learning Framework for Evaluating and Ranking Omics Layers in Cancer Drug Response Prediction
Source: Curr Issues Mol Biol. 2026 Sep 2;48(9):897. doi: 10.3390/cimb48090897 (PMC13606002; doi:10.3390/cimb48090897)
Supplement: Supplementary file 1 [file cimb-48-00897-s001.zip › Table S2.pdf]

Table S2. Clustering Algorithms with Silhouette Scores for Each Cluster, Overall Silhouette Scores and Davies–Bouldin Index.

**Clustering Algorithms**	**Cluster 0**	**Cluster 1**	**Cluster 2**	**Cluster3**	**Cluster 4**	**Overall Silhouette Score**	**Overall Davies–Bouldin Index**
K-Means	0.373375	0.147529	0.072353	0.141592	0.153591	0.170057	1.854821
Agglomerative Clustering	0.033477	0.134035	0.091886	0.382595	0.144694	0.146592	2.033370
K-Means++	Same as K-Means						
Mini-Batch K-Means	0.354509	0.125109	0.155791	0.100698	0.142356	0.169679	1.823567
Spectral Clustering	0.037511	0.377002	0.146907	0.131316	0.185629	0.160152	1.909886
Gaussian Mixture Model (GMM)	0.377402	0.157415	0.050497	0.089663	0.113961	0.146520	2.043284
BIRCH (Balanced Iterative Reducing and Clustering using Hierarchies)	0.033477	0.134035	0.091886	0.382595	0.144694	0.146592	2.033370
Affinity Propagation Clustering	Generated 38 Clusters with overall Silhouette Score					0.093590	2.012433

Table S2. Represents silhouette score values as well as overall silhouette score and overall Davies–Bouldin Index for clusters generated using K (number of cluster) =5, for different clustering algorithms. Best cluster silhouette score for K-Means is better than Mini-Batch K-Means, Affinity Propagation Clustering and same as K-Means++. Agglomerative Clustering, Spectral Clustering, Gaussian Mixture Model (GMM) and BIRCH (Balanced Iterative Reducing and Clustering using Hierarchies) have higher best cluster silhouette score but poor overall silhouette score and Davies–Bouldin Index. K-Means was selected considering its overall performance.
