# Supplementary material for: A Systematic Machine Learning Framework for Evaluating and Ranking Omics Layers in Cancer Drug Response Prediction
Source: Curr Issues Mol Biol. 2026 Sep 2;48(9):897. doi: 10.3390/cimb48090897 (PMC13606002; doi:10.3390/cimb48090897)
Supplement: Supplementary file 1 [file cimb-48-00897-s001.zip › Table S3.pdf]

Table S3. Silhouette Scores and Cluster Sizes for Different Values of K in Clustering using K-Means.

K	C0 (Score, Elements)	C1 (Score, Elements)	C2 (Score, Elements)	C3 (Score, Elements)	C4 (Score, Elements)	C5 (Score, Elements)	C6 (Score, Elements)	C7 (Score, Elements)	C8 (Score, Elements)	Overall Score
2	0.3135, 133	0.2332, 452	-	-	-	-	-	-	-	0.2515
3	0.3449, 119	0.1513, 230	0.1394, 236	-	-	-	-	-	-	0.1859
4	0.3345, 116	0.1523, 131	0.1172, 202	0.1402, 136	-	-	-	-	-	0.1735
5	0.3734, 95	0.1475, 113	0.0724, 112	0.1416, 122	0.1536, 143	-	-	-	-	0.1701
6	0.3397, 94	0.1349, 112	0.0974, 80	0.1387, 110	0.1412, 125	0.1072, 64	-	-	-	0.1617
7	0.3411, 94	0.0954, 87	0.0932, 78	0.1223, 91	0.1405, 119	0.1003, 64	0.1033, 52	-	-	0.1492
8	0.3357, 94	0.0895, 89	0.1023, 62	0.1067, 76	0.1311, 113	0.0587, 52	0.1211, 44	0.0928, 55	-	0.1406
9	0.1057, 34	0.0894, 89	0.1023, 62	0.1067, 76	0.1311, 113	0.0587, 52	0.1211, 44	0.0857, 55	0.1743, 60	0.1100

Table S3. Represents silhouette score and cluster size for different values of K (number of Clusters). K=5 gave highest silhouette score cluster (cluster 0). The value of K was selected considering the need of framework to analyse high-quality cluster. The distribution of cluster elements is better for k=5 compared to k=2 to 4, which also supports selection of k=5 for clustering even when its overall silhouette score is not better than k =2 to 4.
