# Supplementary material for: A Systematic Machine Learning Framework for Evaluating and Ranking Omics Layers in Cancer Drug Response Prediction
Source: Curr Issues Mol Biol. 2026 Sep 2;48(9):897. doi: 10.3390/cimb48090897 (PMC13606002; doi:10.3390/cimb48090897)
Supplement: Supplementary file 1 [file cimb-48-00897-s001.zip › Table S4.pdf]

Table S4. Performance of ML Algorithms Across Clusters for Drug Sensitivity Prediction.

**ML Algorithms**		**Cluster 0**	**Cluster 1**	**Cluster 2**	**Cluster 3**	**Cluster 4**
Random Forest Classifier (RFC)	Mean Accuracy	0.83	0.82	0.81	0.83	0.85
	No. of Drugs with accuracy above 75%	39	28	30	25	17
Support Vector Classifier (SVC)	Mean Accuracy	0.81	0.81	0.77	0.82	0.88
	No. of Drugs with accuracy above 75%	24	21	6	17	4
Gradient Boosting Classifier	Mean Accuracy	0.81	0.82	0.82	0.83	0.85
	No. of Drugs with accuracy above 75%	24	21	21	13	13
AdaBoost Classifier	Mean Accuracy	0.80	0.82	0.80	0.85	0.86
	No. of Drugs with accuracy above 75%	24	12	17	18	9
XGBoost Classifier	Mean Accuracy	0.81	0.83	0.81	0.83	0.86
	No. of Drugs with accuracy above 75%	34	15	21	16	13
Logistic Regression Classifier (LRC)	Mean Accuracy	0.82	0.83	0.80	0.83	0.86
	No. of Drugs with accuracy above 75%	30	23	19	19	5

Table S4. Represents framework's predictive performance using different predictive algorithms. Mean accuracy is the mean of prediction accuracy of drugs with accuracy above 75%. The green-colored cells represent execution in which highest silhouette score cluster gave the best results for drug response prediction. Match percentage was calculated by dividing the number of rows with green colour cell by the total number of rows and multiplying by 100.
